# Associations between physical size and space are strongly asymmetrical

**DOI:** 10.1038/s41598-023-43313-5

**Published:** 2023-09-27

**Authors:** Melanie Richter, Peter Wühr

**Affiliations:** Department of Psychology, TU Dortmund University, Emil-Figge Straße 50, 44227 Dortmund, Germany

**Keywords:** Psychology, Human behaviour

## Abstract

The spatial–size association of response codes (SSARC) effect describes the phenomenon that left responses are faster and more accurate to small stimuli whereas right responses are faster and more accurate to large stimuli, as compared to the opposite mapping. The effect indicates associations between the mental representations of physical size and space. Importantly, the theoretical accounts of SSARC effects make different predictions about the reciprocity and/or symmetry of spatial–size associations. To investigate the reciprocity of SSARC effects, we compared compatibility effects in two verbal choice-response tasks: a size–location (typical SSARC) task and a location–size (reciprocal SSARC) task. In the size–location task, participants responded verbally to a small/large stimulus by saying “left”/“right”. In the location–size task, participants responded verbally to a left-/right-side stimulus by saying “small”/“large”. Participants completed both tasks with a compatible (small–left, large–right; left–small, right–large) and an incompatible (small–right, large–left; left–large, right–small) mapping. A regular SSARC effect emerged in the size–location task. However, no reciprocal SSARC effect emerged in the location–size task if outliers were excluded. If outliers were not excluded, small reciprocal SSARC effects occurred. Associations underlying the SSARC effect are thus strongly asymmetrical: Physical (stimulus) size can prime spatial responses much more strongly than spatial (stimulus) position can prime size-related responses. The finding of asymmetrical associations between size and space is in line with some theoretical accounts of the SSARC effect but at odds with others.

## Introduction

*Stimulus–response compatibility* (*S–R compatibility*) describes the observation that certain assignments between stimulus and response alternatives allow for better performance, i.e., faster responses and higher accuracy, than other assignments^1,2^. The performance difference between such “compatible” and “incompatible” mappings is called *compatibility effect*. Compatibility effects are a widely studied phenomenon in cognitive research because they provide insights into the selection and execution of actions by revealing underlying associations between different stimulus and response dimensions^2,3^. Moreover, those insights can also be of use in applied research, in particular in the field of human factors engineering^4,5^.

One example for S–R compatibility is the compatibility effect between physical stimulus size and spatial response position. The so-called *spatial–size association of response codes* (*SSARC*) *effect* denotes the observation that left responses are faster and more accurate to physically small stimuli whereas right responses are faster and more accurate to physically large stimuli, as compared to the opposite mapping^6–9^. While the spatial–quantity association of response codes (SQUARC) effect refers to compatibility effects between any kind of quantity (height, weight, loudness, luminance) on the stimulus level and spatial responses, the SSARC effect refers to a compatibility effect between physical size in particular and spatial responses^10,11^.

The SSARC effect thus provides evidence for the existence of associations between the mental representations of physical size and space, so-called *spatial–size associations*. Further studies revealed that the SSARC effect also occurs with physical size as a task-irrelevant stimulus feature providing evidence that size is automatically processed and subsequently associated with spatial position^9,12^. Moreover, Wühr et al.^13^ observed that SSARC effects also emerge with different response modalities such as verbal responses. The independence of SSARC effects from response sets implies that the associations between size and space, which underlie the effect, seem to rest upon an intermediate representational level instead of direct relations between stimulus and response codes. Insights from the SSARC effect might also be of use in human factors engineering, which involves, for example, the creation of work environments. Designing manufacturing lines in such a way that small and large items are placed to the left and right of employees, respectively, might for example conform to humans’ automatic spatial–size associations and thus reduce cognitive conflict.

The SSARC effect thus illustrates that physical stimulus size influences spatial responses in such a way that physically small stimuli facilitate the selection and execution of left responses whereas physically large stimuli facilitate the selection and execution of right responses. However, it is unclear if this effect can also occur in the opposite direction with spatial position as relevant stimulus feature and physical size as relevant response feature. The present study therefore investigates if the underlying associations between size and space are reciprocal or not by investigating if spatial stimuli can influence the selection and execution of verbal responses referring to physical sizes. Most interestingly, the theories that have been proposed to account for SSARC effects differ in whether they predict uni- or bidirectional SSARC effects and in whether they predict symmetrical or asymmetrical associations between physical size and space.

The *polarity correspondence principle* proposed by Proctor and colleagues assumes that in many binary classification tasks in which stimuli and responses vary on bipolar dimensions, one stimulus and response alternative is encoded as positive polarity whereas the opposite stimulus and response alternative is encoded as negative polarity^14^. Corresponding stimulus and response polarities lead to faster and more accurate responses than non-corresponding polarities^14–16^. For example, in a typical SSARC task, the categories “small” and “left” are assigned negative polarity whereas the categories “large” and “right” are assigned positive polarity. According to the polarity correspondence principle, SSARC effects should symmetrically emerge in both the regular and the reciprocal direction because opposing alternatives are encoded as negative or positive polarity regardless of whether they vary as stimulus or response feature.

The *working memory* (*WM*) account, which has originally been proposed by van Dijck and colleagues to account for compatibility effects between number and space, assumes that the serial order in which stimuli of a given set are stored in WM corresponds with spatial position. Accordingly, early serial positions are associated with left positions in space, while late serial positions are associated with right positions in space^17,18^. The WM account could explain SSARC effects if one assumes that a set of stimuli, which vary in size during an experiment, is (spontaneously) stored in an ascending order in WM. As a result, small stimuli at early serial positions could prime left responses, whereas large stimuli at late serial positions could prime right responses. However, it is debatable if the WM account predicts reciprocal SSARC effects. The WM account might assume that spatial stimuli, which vary in horizontal location, are stored in a canonical order (i.e., from left to right) in WM, but it remains unclear how the spatial links of serial stimulus positions (early-left, late-right) could then prime non-spatial “size” responses. A possible extension of the WM account might assume that not only sets of stimuli varying in size are stored in an ascending order, but sets of responses varying in (or referring to) size are also stored in an ascending order in WM. As a result, left stimuli would prime (“small”) responses at early serial positions, and right stimuli would prime (“large”) responses at late serial positions.

The so-called *correlations in experience* (*CORE*) *principle* proposed by Pitt and Casasanto^19^ postulates that “people spatialize abstract domains in their minds according to the ways those domains are spatialized in their experience” (p. 1048). Compatibility effects between two dimensions thus arise because they are correlated in people’s natural or cultural world and transferred to their mental representation accordingly. Wühr et al.^13^ observed that handedness and effector strength contribute to the origin of the SSARC effect. In line with the CORE principle, they proposed that the people’s habit to grasp smaller and lighter objects with their weaker non-dominant hand and to grasp larger and heavier objects with their stronger dominant hand determines the associations between physical size and space. Grasping habits consistently involve physical size as stimulus feature and spatial position as response feature and it seems difficult to think of other natural or cultural experiences that might shape spatial–size associations in the reciprocal direction. The account of SSARC effects provided by Wühr et al.^13^ should therefore predict unidirectional or at least strongly asymmetrical associations between size and space.

So far, the question of reciprocity has only been addressed with regards to the so-called *spatial–numerical association of response codes* (*SNARC*) *effect*. The SNARC effect refers to the finding that left responses are faster and more accurate to small numbers whereas right responses are faster and more accurate to large numbers, as compared to the opposite mapping^20–22^. Until now, the question of the origin of spatial–numerical associations has not been finally resolved. While several studies have provided evidence that cultural experiences such as reading direction^23–25^, finger counting habits^26,27^ or visuo-motor activities in general^28^ may shape spatial-numerical associations, a spatial representation of numbers has already been found in pre-school children^29,30^ and even preverbal infants^31,32^ pointing towards a more fundamental origin of spatial–numerical associations. This biological basis of the SNARC effect has also been corroborated by studies revealing that new-born chickens^33^, rhesus monkeys^34^ and honeybees^35^ map numbers onto space.

With human subjects, several studies have demonstrated the bidirectionality of spatial–numerical associations as Stimulus-Stimulus congruency effects in priming tasks^36,37^ and as Response-Response effects in random number generation tasks^38–40^. Nevertheless, spatial–numerical associations in S–R priming tasks appear to be at least strongly asymmetrical: In a previous study^41^, we compared compatibility effects in a number-location task (numerical stimuli, spatial keypress responses), which represents a typical SNARC task, to compatibility effects in a location-number task (spatial stimuli, numerical keypress responses), which represents a reciprocal SNARC task. While we observed regular SNARC effects, we did not observe reciprocal SNARC effects if outlier datasets were excluded. Including outlier datasets led to small reciprocal SNARC effects driven by the small subsample which showed very large reaction times and/or error percentages^41^.

Even though previous studies have thus observed that spatial–numerical associations are strongly asymmetrical, several dissociations between the SNARC and the SSARC effect suggest different underlying origins or mechanisms, and thus prevent generalizing findings and conclusions across both effects. First, the generation of response codes is strongly influenced by external spatial coding in the SNARC effect but by anatomical-based coding in the SSARC effect^42^. Second, handedness does not influence SNARC effects, whereas it does affect SSARC effects^13^. Third, Vellan and Leth-Steensen^7^ showed that SNARC and SSARC effects do not emerge simultaneously even if conditions for both effects were provided. Different mechanisms underlying the SNARC and SSARC effect thus preclude conclusions about the reciprocity of SSARC effects. To our knowledge, this study is the first to investigate if spatial–size associations are reciprocal or not.

To investigate if the associations between physical size and space, which produce the SSARC effect, are bidirectional or not, we applied a similar design we had employed to examine the reciprocity of the SNARC effect^41^. We therefore compared the compatibility effect in a size–location task, which represents a typical SSARC task, to the compatibility effect in a location–size task, which represents a reciprocal SSARC task. Since responses in the location–size task had to vary in physical size which cannot easily be achieved with manual responses, we employed verbal responses in both tasks. Importantly, Wühr et al.^13^ demonstrated that SSARC effects of similar size can be obtained with manual as well as verbal responses. In the size–location task, participants therefore responded to a small or large stimulus by saying “left”or “right”. In the location–size task, participants responded to a left or right stimulus by saying “small” or “large”. Note that participants responded with the German words for “left”, “right”, “small” and “large” in our experiments even though we are using the English words in the text for clarity. Three different patterns of associations between size and space are conceivable. Significant SSARC effects in both directions of similar size would indicate bidirectional and symmetrical associations. Significant SSARC effects in both directions of different size would indicate bidirectional but asymmetrical associations. Significant SSARC effects in the regular but non-significant SSARC effects in the reciprocal direction, however, would point towards unidirectional associations between physical size and space.

## Methods

The experiment was preregistered on the website OpenScienceFramework (OSF) (https://osf.io/sz6ub).

### Participants

In a previous study^41^, we investigated the reciprocity of the SNARC effect and observed a strong main effect of mapping (η^2^_p_ = 0.20), and a strong two-way interaction between task and mapping (η^2^_p_ = 0.19). Hence, for the present experiment, we assumed a η^2^_p_ of 0.20 for the main effect of mapping and for the two-way interaction. We used the software MorePower^43^ for conducting a power analysis, which revealed that a sample size of 54 participants would be required to detect an effect of this size with high power (1 − beta = 0.95) at the standard 0.05 alpha error probability. In order to account for the exclusion of outlier data sets from our analysis, we planned to test a few more than 54 participants.

Fifty-nine volunteer students (56 female, 3 male) with a mean age of 21.695 years (*SD* = 3.007) participated in our experiment and received either course credit or a payment of 10 Euro in exchange. All participants reported to have normal (*N* = 36) or corrected-to-normal (*N* = 23) vision. According to self-report, 52 participants were right-handed, whereas the remaining seven participants were left-handed. Prior research has shown that handedness modulates the SSARC effect^13^. We decided to nevertheless include left-handed participants in our sample for two reasons. Firstly, there was only a small number of seven left-handed participants in our sample. Secondly, and more importantly, even though handedness modulates the SSARC effect, it merely weakens but does not reverse the effect. In other words, left-handed participants do show similar but smaller SSARC effects as right-handed participants^13^. Prior to participation, volunteers gave their informed consent. The local Ethics Committee at TU Dortmund University approved the experimental protocol for our study (GEKTUDO_2022_36). We confirm that all methods were performed in accordance with the relevant guidelines and regulations.

### Apparatus and stimuli

With a viewing distance of approximately 50 cm, participants sat in front of a customary 19-inch color monitor. The software EPrime 3.0 (Psychology Software Tools; Sharpsburg, PA, USA) controlled the presentation of stimuli and registered responses (i.e., verbal response, reaction time (RT)). A small plus sign (Courier font, size 18 pt), which was presented at the screen center at the beginning of each trial, served as a fixation point. All imperative stimuli were presented in black on a white background. In the size–location task, the imperative stimulus was one small (side length = 2 cm) or one large (side length = 4 cm) filled square presented at screen center (position: X = center, Y = center). Participants responded verbally by saying “left” or “right” into a microphone which was placed in front of them and centrally aligned to their midline. To register RTs and record the participants’ verbal responses, the microphone was connected to the voice-key of the Chronos console (Psychology Software Tools; Sharpsburg, PA, USA). Each vocal response was stored in a sound file for a later check of its accuracy. In the location–size task, the imperative stimulus was a black square with a side length of 2 cm that appeared at the center of the left (position: X = 25%, Y = center) or the right screen half (position: X = 75%, Y = center). Participants responded verbally by saying “small” or “large”.

### Procedure

**Figure 1:**
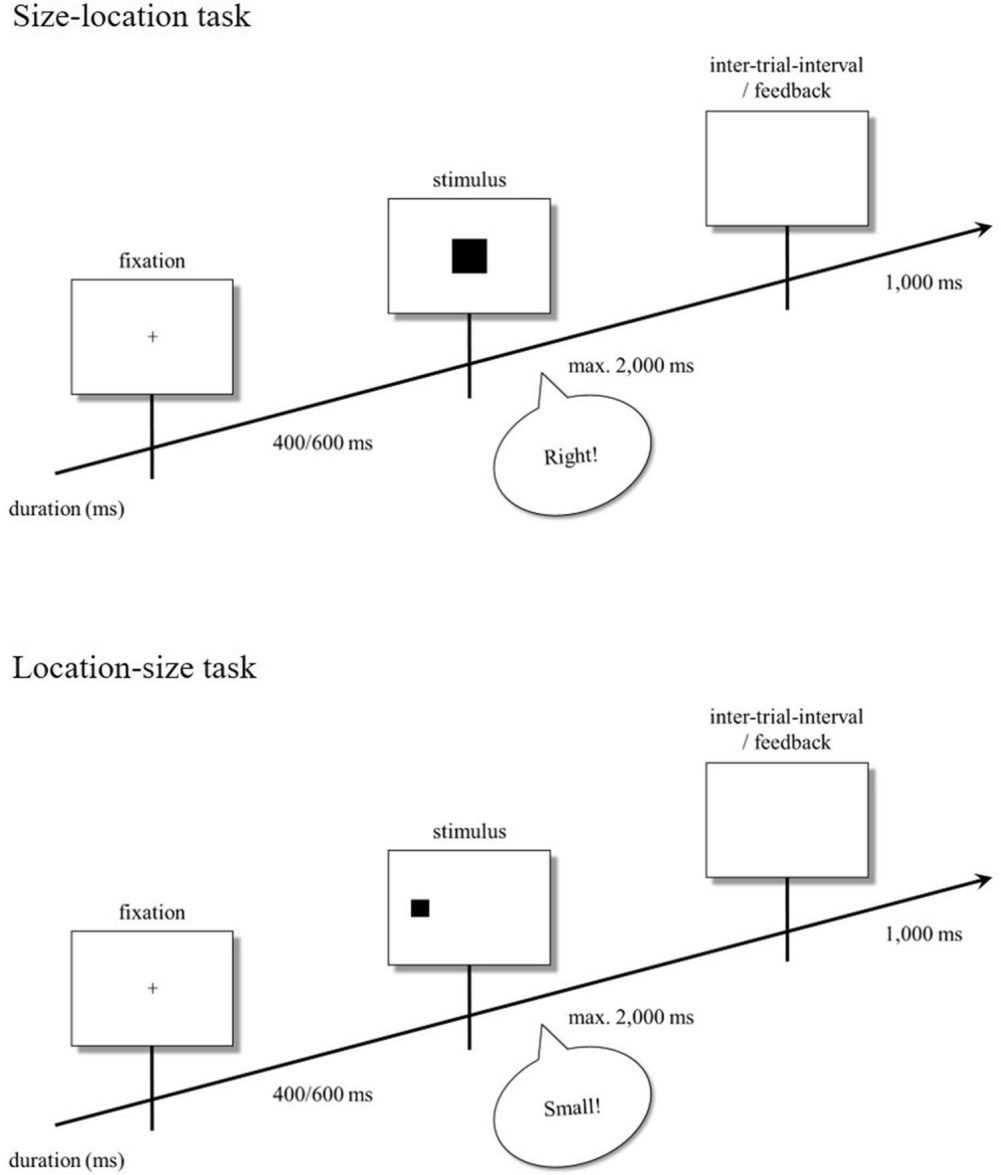
Time course of events in typical trials of the size–location task (upper panel), and the location–size task (lower panel) according to compatible mappings. Feedback was only provided after a missing response.

The orthogonal combination of two tasks (size–location task, location–size task) and two S–R mappings (compatible, incompatible) resulted in four conditions which were completed by each participant. In the regular size–location task, participants verbally responded to stimulus size (small or large) by saying “left” or “right” according to a compatible mapping (small–left, large–right) or an incompatible mapping (small–right, large–left). In the location–size task, participants verbally responded to stimulus location (left or right) by saying “small” or “large” according to a compatible mapping (left–small, right–large) or an incompatible mapping (left–large, right–small). The time course and sample stimuli of the size–location task and the location–size task are depicted in Fig. 1.

Instructions presented at the beginning of each condition informed participants about the content and the procedure of the following task. Each condition consisted of one training block containing 10 trials and two experimental blocks containing 40 trials each. We randomized trials within blocks. Each trial started with the presentation of a fixation point for 400 or 600 ms, with both durations occurring equally often within each block. Subsequently, the imperative stimulus was presented until a response was recorded or for a maximum of 2000 ms. An inter-trial interval with an empty screen was presented for 1000 ms after a response was given whereas a corresponding error message was presented during the inter-trial interval after a missing response. Participants were not provided feedback about response accuracy because the program could not determine the correctness of verbal responses. At the beginning of each experimental block, task and S–R mapping instructions were repeated. Participants were able to take a break between blocks or to continue with the subsequent one.

The experiment took about 30–40 min. The experimenter left the laboratory before participants started the experimental blocks. The order of tasks (size–location or location–size first) and the order of mappings (compatible or incompatible mapping first) were counterbalanced between participants. Participants completed both S–R mapping conditions consecutively within one task and the order of mappings was held constant between tasks within one participant.

### Design and data analysis

For each participant, the verbal responses recorded in each trial were checked for accuracy, and response errors were manually entered into the data file before the statistical analysis. The experimental design was a two-factorial (*Task *× *Mapping*) within-subjects design. The factor *Task* had two levels: the size–location task with size (small vs. large) as the critical stimulus feature and location (left vs. right) as the critical response feature and the location–size task with location (left vs. right) as the critical stimulus feature and size (small vs. large) as the critical response feature. The factor *S–R Mapping* also had two levels: a compatible mapping (small–left, large–right in the size–location task; left–small, right–large in the location–size task) and an incompatible mapping (small–right, large–left in the size–location task; left–large, right–small in the location–size task). Reaction Times (RTs) of correct verbal responses and error percentages served as dependent variables.

With a two-way ANOVA, we planned to investigate the impact of the two independent variables (i.e., Task, Mapping) on the dependent variables (i.e., RTs, error percentages). In case of a significant two-way interaction, we planned to conduct *t* tests to determine the source of the interaction. Even though error percentages are typically not normally distributed, we preferred using *t* tests instead of non-parametric tests because error results often contain a large number of ties, which provide evidence for H0, but are excluded from non-parametric tests and thus bias the results. Since the assumption of unidirectional associations between size and space predicts a null effect in the location–size task, we needed to evaluate the evidence for H1 and H0 likewise and thus reported the Bayes Factor (BF) for each pairwise comparison^44^. We used the evidence categories provided by Jeffreys, 1961 (as cited in Lee and Wagenmakers^45^) to interpret the BF values.

Moreover, we conducted a distributional analysis investigating the time course of the SSARC effect in both the regular and the reciprocal direction. This was motivated by two objectives: firstly, previous studies have shown that response speed (i.e., RT) determines the size of compatibility or congruency effects^46^. For example, it has been shown that both SNARC effects^47^ and SSARC effects with manual responses^48^ increase in size with increasing RTs. We therefore investigated the distribution of SSARC effects to, firstly, specify the time course of SSARC effects with verbal responses. Secondly, we aimed to detect small mapping effects which might have emerged in the reciprocal direction for specific RT levels only without reaching significance in the omnibus analysis. We applied Ratcliff’s method of vincentizing^49^ to analyze the time course of the mapping effects. For each participant and condition, we divided the rank-ordered RTs into four quartiles and computed the corresponding means, which we then subjected to a three-factorial ANOVA with Task (size–location task, location–size task), Mapping (compatible, incompatible) and Quartile (1–4) as within-subject variables.

### Ethics approval

The local Ethics Committee at TU Dortmund University had approved the experimental protocol for our study (approval no. GEKTUDO_2022_36). We confirm that all methods were performed in accordance with the relevant guidelines and regulations.

### Informed consent

Before the experiment, all participants gave written informed consent to participate.

## Results

### Data trimming

We excluded three participants (participant numbers 4, 8 and 22 in the dataset) from data analysis because their mean error percentage exceeded 20% in one of the two tasks. Excluding these datasets reduced the highest error percentages to 11.5% in the size–location task, and 3.2% in the location–size task. Our remaining sample thus included 56 participants. In less than 1% of trials in both the size–location (*M* = 0.096%, *SD* = 0.399) and location–size task (*M* = 0.198%, *SD* = 0.768), participants’ responses were too fast (i.e., RT < 100 ms). Likewise, in less than 1% of trials in both the size–location (*M* = 0.344%, *SD* = 0.930) and location–size task (*M* = 0.116%, *SD* = 0.410) participants’ responses were too slow (i.e., RT > 1500 ms). We excluded these trials with RTs below 100 ms or above 1500 ms as well as the first trial in each block from data analysis.

### Reaction times (RTs)

In a preliminary (not preregistered) analysis, we tested for possible effects of *Task Order* on the effects of *Task* (size–location task vs. location–size task) and *Mapping* (compatible vs. incompatible) on RTs. Note that the degrees of freedom for this analysis were different from analyses without the order variable, because the number of excluded cases was different for the two order conditions. A three-factorial Analysis of Variance (ANOVA) neither revealed a main effect of *Task Order*, *F*(1, 54) = 1.440, *MSE* = 19,944, *p* = 0.235, η^2^_p_ = 0.026, nor any interactions between *Task Order* and the other factors, all *F*(1, 54) < 1.0, all *p* > 0.450, all η^2^_p_ < 0.01.

A two-factorial ANOVA, with *Task* and *Mapping* as within-subject factors, revealed two significant main effects and a significant two-way interaction. The significant main effect of *Task*, *F*(1, 55) = 128.230, *MSE* = 1545.239, *p* < 0.001, η^2^_p_ = 0.700, reflected shorter RTs in the location–size task (*M* = 410 ms, *SD* = 76) than in the size–location task (*M* = 469 ms, *SD* = 77). The significant main effect of *Mapping*, *F*(1, 55) = 15.207, *MSE* = 893.662, *p* < 0.001, η^2^_p_ = 0.217, indicated shorter RTs with the compatible mapping (*M* = 432 ms, *SD* = 80) than with the incompatible mapping (*M* = 447 ms, *SD* = 84). Crucially, however, the significant two-way interaction, *F*(1, 55) = 10.192, *MSE* = 803.552, *p* = 0.002, η^2^_p_ = 0.156, revealed different mapping effects in the two tasks.

**Figure 2:**
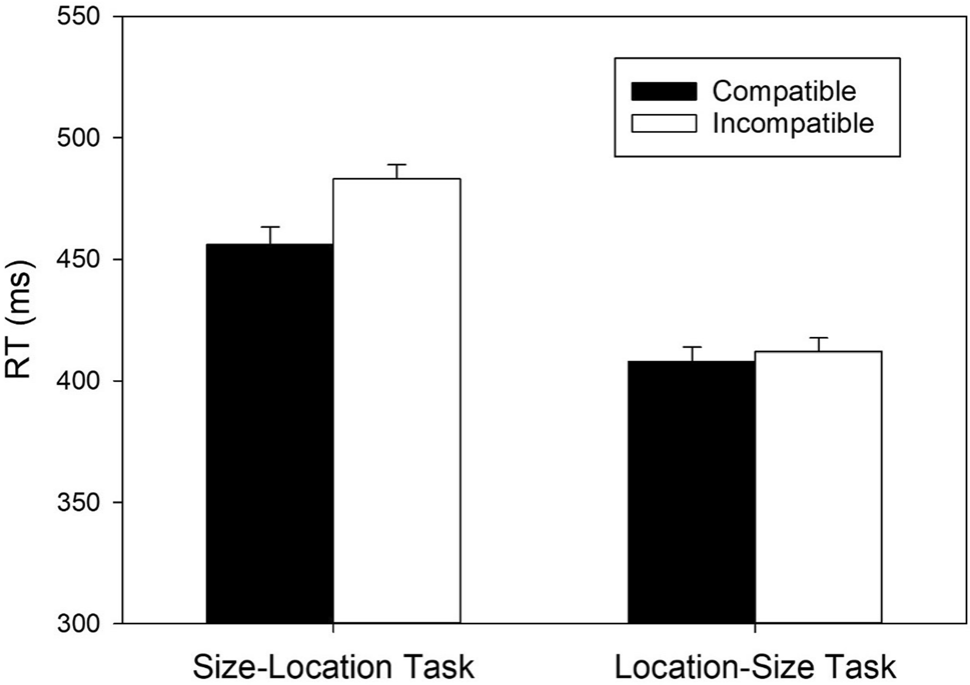
RTs of correct responses as a function of Task and S–R mapping (*N* = 56). Error bars reflect 95% confidence intervals for within-subjects designs^50^.

We conducted pairwise comparisons between compatible and incompatible mappings for each task to determine the source of the two-way interaction. In the size–location task, RTs were significantly shorter in the compatible than in the incompatible condition, *t*(55) = 4.421, *p* < 0.001, *d* = 0.591, BF_+0_ = 447.399, revealing a regular SSARC effect of 28 ms (cf. Fig. 2) and extreme evidence for the presence of a mapping effect. In contrast, in the location–size task, RTs did not differ significantly between the two mapping conditions, *t*(55) = 0.753, *p* = 0.455, *d* = 0.101, BF_+0_ = 0.191, (cf. Fig. 2) reflecting moderate evidence against the presence of a reciprocal SSARC effect.

### Error percentages

Overall error percentages were very low thus limiting the interpretability of the statistical analysis. Even though we report the results for the sake of completeness, the results should therefore be interpreted with caution. A potential speed-accuracy trade-off, however, can be ruled out as error percentages were the highest in the task in which RTs were the slowest.

In a preliminary (not preregistered) analysis, we tested for possible effects of *Task Order* on the effects of *Task* and *Mapping* on error percentages. A three-factorial ANOVA showed a significant effect of *Task Order*, *F*(1, 54) = 9.430, *MSE* = 6.790, *p* = 0.003, η^2^_p_ = 0.149, but no significant interaction between *Task Order* and the other factors, all *F*(1, 54) < 3.50, all *p* > 0.050, all η^2^_p_ < 0.070. The main effect of task order reflected more errors (across both tasks) when the location–size task was done first (*M* = 1.835, *SD* = 3.133) than when the size–location task was done first (*M* = 0.763, *SD* = 1.490).

A two-factorial ANOVA, with *Task* and *Mapping* as within-subjects factors, also revealed two significant main effects and a significant two-way interaction. The significant main effect of *Task*, *F*(1, 55) = 19.255, *MSE* = 4.894, *p* < 0.001, η^2^_p_ = 0.259, reflected more errors in the size–location task (*M* = 1.909, *SD* = 3.173) than in the location–size task (*M* = 0.612, *SD* = 1.072). The significant main effect of *Mapping*, *F*(1, 55) = 8.642, *MSE* = 5.644, *p* = 0.005, η^2^_p_ = 0.136, indicated less errors with the compatible mapping (*M* = 0.794, *SD* = 1.471) than with the incompatible mapping (*M* = 1.727, *SD* = 3.076). Crucially, however, the significant two-way interaction, *F*(1, 55) = 4.286, *MSE* = 3.140, *p* = 0.043, η^2^_p_ = 0.072, again revealed different mapping effects in the two tasks.

**Figure 3:**
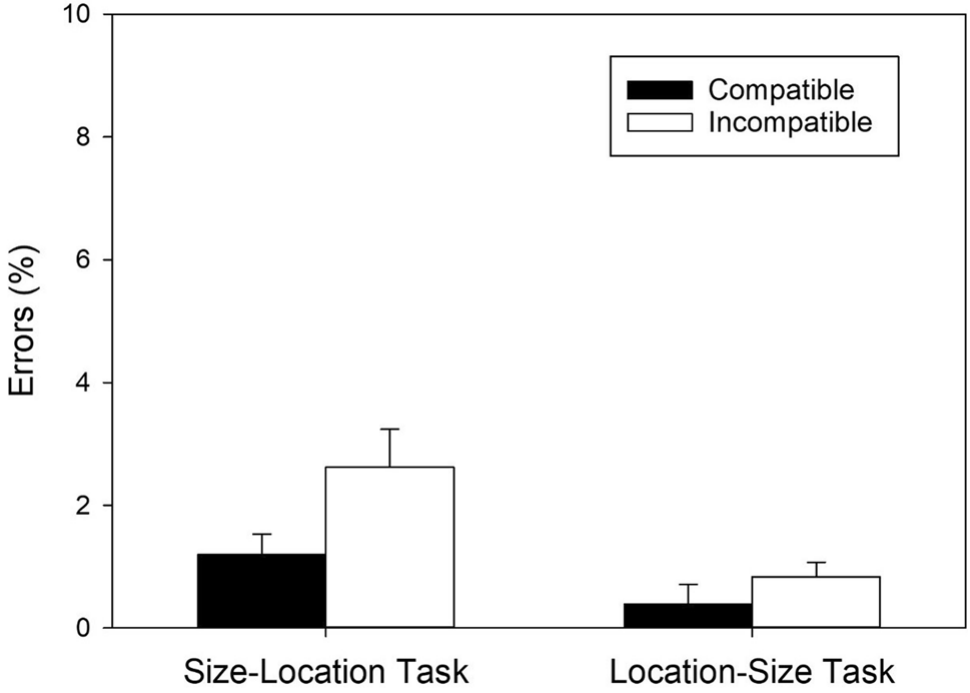
Error percentages as a function of Task and S–R mapping (*N* = 56). Error bars reflect 95% confidence intervals for within-subjects designs^50^.

In the size–location task, errors were significantly less frequent in compatible than in incompatible conditions, *t*(55) = 2.692, *p* = 0.009, *d* = 0.360, BF_+0_ = 3.797, revealing a regular SSARC effect of 1.424% (cf. Fig. 3) and moderate evidence for the presence of a mapping effect. Similarly, in the location–size task, errors were also significantly less frequent in compatible than in incompatible conditions, *t*(55) = 2.401, *p* = 0.020, *d* = 0.321, BF_+0_ = 2.014, revealing a reciprocal SSARC effect of 0.443% (cf. Fig. 3) and anecdotal evidence for the presence of a mapping effect.

### Distributional analysis for RTs

**Figure 4:**
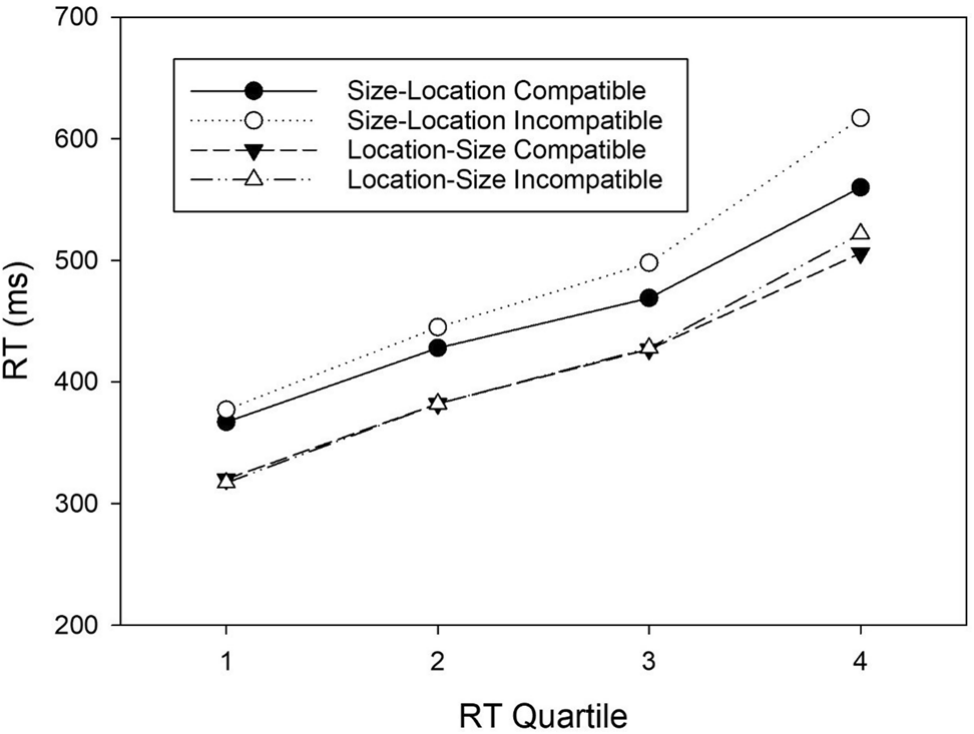
RTs of correct responses as a function of Task, S–R mapping, and RT quartile (*N* = 56).

We conducted a three-factorial ANOVA with *Task* (size–location task, location–size task), *Mapping* (compatible, incompatible) and *Quartile* (1–4) as within-subject variables and RT means as the dependent variable. Figure 4 illustrates the corresponding means. Note that we will only report results of interest, which are the interactions between *Quartile* and the other variables.

Both two-way interactions *Quartile *× *Task* and *Quartile* × *Mapping*, and the three-way interaction were significant. The significant *Task* × *Quartile* interaction, *F*(3, 165) = 11.179, *MSE* = 477.614, *p* < 0.001, η^2^_p_ = 0.169, revealed that, with increasing RTs, mean RTs in the size–location task became increasingly slower compared to the location–size task. The range of RTs was thus smaller in the latter compared to the former task. The significant *Mapping* × *Quartile* interaction, *F*(3, 165) = 22.577, *MSE* = 524.907, *p* < 0.001, η^2^_p_ = 0.291, indicated that mapping effects increased with increasing RTs. Crucially, however, the significant three-way interaction, *F*(3, 165) = 5.839, *MSE* = 382.568, *p* < 0.001, η^2^_p_ = 0.096, revealed that the time course of the mapping effects differed between both tasks.

To determine the source of the significant three-way interaction, we conducted a 2 × 4 ANOVA, with *Mapping* and *Quartile* as within-subject variables, for each task separately. In the size–location task, the main effect of *Mapping*, *F*(1, 55) = 19.790, *MSE* = 4413.287, *p* < 0.001, η^2^_p_ = 0.265, as well as the *Mapping *× *Quartile* interaction, *F*(3, 165) = 20.969, *MSE* = 576.469, *p* < 0.001, η^2^_p_ = 0.276, were significant. The two-way interaction indicated that the mapping effect increased with increasing RT level. More specifically, the mapping effect increased from 9 ms in the first quartile, to 17 ms in the second quartile, 28 ms in the third quartile, and 57 ms in the fourth quartile. Post-hoc tests between the compatible and incompatible mapping condition for each quartile indicated that the regular SSARC effect was non-significant in the first quartile, *t*(55) = 1.927, *p*_*Tukey*_ = 0.539, but significant in all three larger quartiles, all *t*s ≥ 3.466, all *p*s_*Tukey*_ ≤ 0.022.

In the location–size task, the main effect of *Mapping*, *F*(1, 55) = 0.546, *MSE* = 2446.171, *p* = 0.463, η^2^_p_ = 0.010, was non-significant, reflecting the absence of a reciprocal SSARC effect. The *Mapping *× *Quartile* interaction, however, *F*(3, 165) = 6.032, *MSE* = 331.005, *p* < 0.001, η^2^_p_ = 0.099, reached significance revealing that the RT difference between the compatible and incompatible condition increased with increasing RT level. More specifically, the mapping effect increased from − 3 ms in the first quartile, to 0 ms in the second quartile, 1 ms in the third quartile, and 16 ms in the fourth quartile. However, post-hoc tests revealed that the reciprocal SSARC effect was non-significant in all four quartiles, all *t*s ≤ 1.922, all *p*s_*Tukey*_ ≥ 0.543.

### Exclusion of outliers

Significant reciprocal SSARC effects emerged in the analysis of error percentages. However, a previous study on the reciprocity of SNARC effects has shown that overall outlier datasets might be driving these reciprocal mapping effects^41^. We therefore decided to exclude outlier participants according to the Tukey criterion^51^ and conduct the same set of analysis once again. According to Tukey^51^, observations below *Q*_*25*_ − 1.5*IQR or above *Q*_*75*_ + 1.5*IQR are classified as outliers. After collapsing data across the mapping variable, we applied the criterion to the remaining four variables (mean RT and error percentage in the size–location and location–size task respectively), according to which we excluded seven further participants (participant numbers 16, 31, 35, 48, 55, 57 and 59). The remaining sample thus consisted of 49 participants. In the analysis of error percentages without outliers, the formerly significant pairwise comparison between the compatible and incompatible condition in the location–size task became non-significant, *t*(48) = 1.453, *p* = 0.153, *d* = 0.208, BF + 0 = 0.415, indicating that error percentages did not differ significantly between the two mapping conditions and providing anecdotal evidence against the presence of a reciprocal SSARC effect when outlier datasets were excluded. Excluding outlier datasets, however, did not affect the pattern of results in the distributional analysis. In the location–size task, the *Mapping *× *Quartile* interaction, *F*(3, 144) = 6.763, *MSE* = 305.274, *p* < 0.001, η^2^_p_ = 0.123, remained significant revealing that the reciprocal mapping effect increased with increasing RT level. More specifically, the mapping effect increased from − 4 ms in the first quartile, to 0 ms in the second quartile, 1 ms in the third quartile, and 16 ms in the fourth quartile. However, post-hoc tests revealed that the reciprocal SSARC effect remained non-significant in all four quartiles, all *t*s ≤ 1.844, all *p*s_Tukey_ ≥ 0.594, when outlier datasets were excluded.

## Discussion

In the present experiment, we investigated if associations between space and physical size, which give rise to the SSARC effect, are reciprocal or not by comparing compatibility effects in a typical size–location task to compatibility effects in a reciprocal location–size task. As expected, we found a regular SSARC effect in the size–location task indicating faster and more accurate left responses to small stimuli and faster and more accurate right responses to large stimuli as compared to the opposite mapping. Interestingly, the regular SSARC effect increased in size with increasing RTs suggesting that the effects of spatial–size associations gradually evolve in the course of response selection and execution. Hence, the SSARC effect with verbal responses shows a similar time course as the SSARC effect with manual responses^48^, and the SNARC effect^47,52^.

In the location–size task, we did not find a significant reciprocal SSARC effect for RTs: RTs did not differ between the compatible (left S—“small”; right S—“large”) and the incompatible mapping condition (left S—“large”; right S—“small”). We did, however, observe a significant reciprocal SSARC effect for error percentages with more accurate responses in the compatible compared to the incompatible mapping condition. Yet, this reciprocal SSARC effect vanished when outlier participants were excluded implying that only a small subsample of participants with very large RTs and/or error percentages showed a reciprocal SSARC effect in error percentages. The distributional analysis of reciprocal SSARC effects revealed a significant interaction between the mapping effect and the quartile in the location–size task which indicated increasing numerical SSARC effects with increasing response time. Even though post-hoc tests demonstrated that no significant reciprocal SSARC effect occurred throughout the entire RT range, the time course pattern of reciprocal SSARC effects thus seems to be similar to the one of regular SSARC effects with the effects of potential associations gradually evolving in the course of response selection and execution.

### Implications for theoretical accounts of SSARC effects

Taken together, we observed that associations between physical size and space are strongly asymmetrical in such a way that the physical size of stimuli influences the selection and execution of spatial responses but that spatial positions of stimuli do not to the same extent influence the selection and execution of responses that vary in physical size. This finding has several implications for the theoretical accounts of the SSARC effect. The polarity correspondence principle, for example, proposes that SSARC effects emerge because the categories “small” and “left” are assigned negative polarity whereas the categories “large” and “right” are assigned positive polarity and corresponding polarities facilitate performance^14–16^. Importantly, in our view, the polarity correspondence principle should predict reciprocal and symmetrical SSARC effects because categories are assigned polarities regardless of whether those categories vary on a stimulus or response level. More precisely, “small” and “left”/“large” and “right” should be encoded as negative/positive polarity both as a stimulus or response feature. Our observation of strongly asymmetrical SSARC effects therefore cannot be explained by the polarity correspondence principle.

According to the WM account, SSARC effects occur because of short-term associations between the serial order in which stimuli of variable size are stored in WM and corresponding spatial positions^17,18^. Assuming that location stimuli are represented in WM in a canonical (i.e., left-to-right) order in the location-size task would not suffice to produce a reciprocal SSARC effect because there is no overlap between serial (or physical) stimulus positions and non-spatial “size” responses. Assuming that not only stimuli of variable size are stored in an ascending order in WM, but responses varying in or referring to different sizes are also stored in an ascending order in WM would render reciprocal SSARC effects possible. The fact that reciprocal SSARC effects were much weaker than the regular SSARC effect in our experiment, hence suggests that participants do not, or rarely, represent responses varying in size in an ascending order in WM.

According to the CORE principle, SSARC effects occur because participants have experienced a systematic relationship between physical stimulus size and response position in their everyday life^19^. Wühr et al.^13^ proposed that the habit to grasp smaller/lighter objects with the weaker non-dominant hand and to grasp larger/heavier objects with the stronger dominant hand is responsible for the spatialization of physical size. Since grasping habits consistently involve physical size as stimulus feature and spatial position as response feature, the account of SSARC effects provided by Wühr et al.^13^ predicts unidirectional or at least strongly asymmetrical associations between size and space. This prediction is compatible with our observation of strongly asymmetrical SSARC effects.

### Comparing reciprocity in SSARC and SNARC effects

This study is the first to investigate if spatial–size associations are reciprocal or not by directly comparing SSARC effects in a size–location and a location–size task. In a previous study^41^, we had used a similar experimental design to investigate the reciprocity of SNARC effects. Most interestingly, the pattern we found in this study for SSARC effects is quite similar to the one we observed for SNARC effects. Spatial–numerical associations in S–R priming tasks also seem to be strongly asymmetrical: While we observed regular SNARC effects with numerical stimuli, we did not observe reciprocal SNARC effects with spatial stimuli, if outlier datasets were excluded. Similar to SSARC effects, including outlier datasets led to small reciprocal SNARC effects driven by a small subsample with very large RTs and/or error percentages^41^. Even though several differences between SSARC and SNARC effects have so far been documented^7,13,42^, the pattern of strongly asymmetrical compatibility effects seems to be a shared characteristic between the SSARC and the SNARC effect. Moreover, in both effects, a small subsample with very large RTs and/or error percentages can produce small reciprocal effects.

### Limitations and avenues for future research

In the present study, we found trends of small reciprocal SSARC effects, however, the circumstances under which they occur are not yet clear. The results of our experiment suggest that two factors might contribute to the occurrence of reciprocal SSARC effects: RT duration and participants’ features. The distributional analysis revealed that reciprocal SSARC effects increased with increasing RTs but did not reach significance in our experiment. This raises the question if prolonged RTs could induce significant reciprocal SSARC effects. Manipulating task demands in such a way that RTs increase might thus form an approach for future research. Moreover, a small subsample of outlier participants with very large RTs and/or error percentages showed reciprocal SSARC effects in error percentages. This raises the question which inter-individual differences are responsible for the occurrence of reciprocal SSARC effects, which so far also remains an issue of future research.

One limitation of our study is that our sample was not balanced in terms of gender. While there is some evidence for an effect of gender on the spatial representation of numbers, which seems to be stronger for male than for female participants^53^, it is unclear if gender affects the SSARC or reversed SSARC effect in a similar manner. However, even if male participants showed a stronger SSARC effect and potentially also a stronger reversed SSARC effect, it seems unlikely that the asymmetry of spatial–size associations vanishes for male participants. Nevertheless, effects of gender on spatial–size representations should be addressed by future research. Furthermore, we can so far primarily conclude the existence of strongly asymmetrical associations between physical size and space for right-handers since our sample consisted of mostly right-handed participants. For left-handers, the asymmetry of spatial–size associations remains to be tested.

## Conclusion

The present experiment demonstrates that the associations between physical size and space which underlie the SSARC effect are strongly asymmetrical: physical (stimulus) size can prime spatial responses much more strongly than spatial (stimulus) position can prime size-related responses. This finding has implications for several theoretical accounts: while the polarity correspondence principle cannot explain asymmetrical associations between size and space, the finding of asymmetrical spatial–size associations is in line with an application of the CORE principle by Wühr et al.^13^.

## Data Availability

The dataset has been published on the “Mendeley Data” repository (https://data.mendeley.com/datasets/b57tbsprzb/1). The audio-files containing participants’ vocal responses can be obtained by contacting the corresponding author (melanie2.richter@tu-dortmund.de). Materials and codes used in this study can also be obtained by contacting the corresponding author (melanie2.richter@tu-dortmund.de).
